# Functional improvement assessed by multifocal electroretinogram after Ocriplasmin treatment for vitreomacular traction

**DOI:** 10.1186/s12886-016-0284-3

**Published:** 2016-07-18

**Authors:** Settimio Rossi, Francesco Testa, Paolo Melillo, Ada Orrico, Michele Della Corte, Francesca Simonelli

**Affiliations:** Eye clinic, Multidisciplinary Department of Medical, Surgical and Dental Sciences, Second University of Naples, Via Sergio Pansini, 5, 80131 Naples, Italy

**Keywords:** Vitreomacular traction, Ocriplasmin, Multifocal electroretinogram, Optical coherence tomography

## Abstract

**Background:**

To evaluate the functional recovery of patients with symptomatic vitreomacular traction (VMT) after Ocriplasmin treatment.

**Methods:**

Prospective, single centre, consecutive case series. Patients were treated with a single intravitreal injection of Ocriplasmin (Jetrea, Thrombogenics Inc, USA, Alcon/Novartis EU). The following outcome measures are considered: resolution of VMT, evaluated through the use of optical coherence tomography (SD-OCT), functional recovery evidenced by multifocal-electroretinogram (mfERG) and microperimetry (MP1) after treatment with Ocriplasmin.

**Results:**

Four eyes of four patients were treated with Ocriplasmin injection. We observed a VMT non-surgical resolution in all patients. The longitudinal statistical analysis showed a significant improvement of best corrected visual acuity (BCVA) in the treated eye of about 0.97 letters/week (*p* = 0.033). No significant difference was observed in mean sensitivity (*p* > 0.05) assessed by MP1 in both eyes, while improvement in fixation stability was assessed in treated eyes (β = 0.39; *p* = 0.029). In the four treated eyes mfERG revealed an increased foveal peak response over the follow-up. The longitudinal analysis of mfERG data shows a significant increase of N1 and P1 amplitude in the first rings and a significant decrease of N1 and P1 implicit time in most rings.

**Conclusions:**

We report on four cases with resolution of VMT after Ocriplasmin treatment. Our preliminary results demonstrate that Ocriplasmin is safe and effective in the treatment of VMT, because it not only leads to a morphological recovery but mostly to a restoration of macular functionality, evaluated through the use of different objective tests, such as MP1 and mfERG over a six-month follow-up.

**Electronic supplementary material:**

The online version of this article (doi:10.1186/s12886-016-0284-3) contains supplementary material, which is available to authorized users.

## Background

The treatment of vitreomacular interface disorders, including symptomatic vitreomacular adhesion (VMA), vitreomacular traction (VMT), and evolving or early macular hole (MH), has traditionally been treated by vitreo-retinal surgery. Recently, Ocriplasmin (Jetrea; Thrombogenics), a recombinant truncated form of human serine protease plasmin with activity against components of the vitreoretinal interface, including fibronectin and laminin, was approved for the treatment of symptomatic VMA [1]. When injected intravitreally, Ocriplasmin induces vitreous liquefaction and separation of vitreoretinal adhesions at the macula and peripapillary retina [2]. In pivotal phase 3 clinical trials, a one-time intravitreal injection of Ocriplasmin (125 μg per 100 μL) was administered in treatment for symptomatic VMA including small (less than or equal to 250 μm) and medium (250 to 400 μm) MH with persistent VMA [1]. When evaluating the pooled data from the pivotal studies, the incidence of non-surgical resolution of VMA was found to be 26.5 %, compared to 10.1 % with placebo injection [1].

Although the safety and clinical efficacy of Ocriplasmin have been established in two phase 3 vehicle-controlled clinical studies, the long-term effects of Ocriplasmin have not been widely documented: recently some small case series and case reports showed visual disturbances and/or adverse events (e.g., acute severe panretinal dysfunction [3], disruption of photoreceptor inner segment–outer segment [4]) after Ocriplasmin injection. The controversial findings of the different studies and the limited number of published cases require further evidence to show the safety profile and effectiveness of Ocriplasmin. Moreover, the studies in the literature analyzed the visual recovery of patients undergoing Ocriplasmin injection by assessing visual acuity, [1, 5–7] full-field electroretinogram (ERG), [3, 4, 8] and morphological recovery on optical coherence tomography (OCT) scans [1, 5–7]. Finally, only two recent clinical cases showed functional findings before and after Ocriplasmin injection evaluated by multifocal electroretinogram (mfERG) [4, 9].

The purpose of the current study is to provide more evidence about safety profile and clinical effectiveness of Ocriplasmin, by reporting a case series of four patients treated by Ocriplasmin injection for symptomatic VMT and MH in our University Hospital. We assessed the effect of treatment by conventional morphologic tests (i.e., OCT). Furthermore, for the first time in the literature, we evaluated macular functionality through the study of tests such as Microperimetry (MP1) and mfERG over a six month follow-up.

## Methods

After the approval of Ocriplasmin for VMT in March 2013, we injected Ocriplasmin in a group of four patients (four eyes). Inclusion criteria were: age over 18 years, adhesion diameter less than 1,500 μm, presence of natural lens, absence of epiretinal membrane, and alteration in visual functionality (i.e., BCVA reduction, metamorphopsia) in the eye to be treated. After dilution with 0.2 mL of sodium chloride 9 mg/mL (0.9 %) solution for injection, 0.1 mL of the diluted solution, containing 125 μg of Ocriplasmin, was injected into the vitreous. All patients received peribulbar anaesthesia and each injection was performed with observation of ocular fundus in order to inject the medication as close as possible to the optic disk. Each patient was examined at baseline and follow-up visits were scheduled at one, two, four weeks, three months and six months. Each visit included best corrected visual acuity (BCVA) measurement, spectral domain optical coherence tomography (SD-OCT) and, from the second week of follow-up, also mfERG and MP1. The study adhered to the tenets of the Declaration of Helsinki and received approval by the Local Ethics Committee of the Second University of Naples. Moreover, each patient gave written informed consent.

BCVA was measured by using ETDRS charts. Metamorphopsia was assessed by Amsler grid test. OCT was performed with Cirrus HD-OCT (Carl Zeiss, Dublin, CA). The acquisition protocol comprised both a five-line raster scan and a macular cube scan pattern (512x128 pixels) in which a 6 x 6-mm region of the retina was scanned within a scan time of 2.4 s. MP1 was performed by an automatic fundus-related perimeter (MP1 Microperimeter, Nidek Technologies, Padova, Italy). The following parameters were used: a fixation target of 2° in diameter consisting of a red ring; a white, monochromatic background with a luminance of 1.27 cd/m^2^; a Goldman III–size stimulus with a projection time of 200 ms; and predefined automatic macular test pattern covering 6° centred onto the gravitational centre of all the fixation points with 43 stimuli. The mfERG responses from the treated eyes of the subjects were performed using VERIS (Version 3, EDI, CA, US) according to the guidelines of the International Society for Clinical Electrophysiology of Vision [10]. The implicit time and amplitude of N1 and P1 waves were computed and analyzed.

Continuous variables are expressed as mean ± standard deviation. A longitudinal analysis of clinical parameters (i.e. BCVA, MP1 parameters and mfERG responses), was performed by repeated measure regression models estimated using Generalized Estimating Equations (GEE), since GEE enable to deal with correlated data (such as longitudinal data) also in small size clinical trials [11–13]. Relationship between BCVA and mfERG response parameter was investigated by regression models estimated by GEE. For the regression models, the correlation coefficients β, which express the mean estimated change of the parameter per week over the follow-up, are provided. A *p*-value less than 5 % was considered statistically significant.

## Results

Table 1 describes the main clinical and demographic parameters of the study subjects. A total of 4 consecutive patients with symptomatic VMT were treated with intravitreal Ocrisplasmin injections by one retina specialist. In one patient VMT was associated with a full-thickness MH, while the remaining three patients had only VMT. The mean age was 67.2 ± 1.3 years (from 64 to 69 years). There were no cases of post-injection uveitis, endophthalmitis, retinal tears, or retinal detachment throughout the follow-up period.

**Table 1:** Clinical and demographic parameters of the included subjects

Id	Age	Gender	Lens status	Meta-morphopsia	Bas. VMA Dia. (μm)	Bas. MH Dia. (μm)	Baseline BCVA	Last (6 months) BCVA	VMT Release	Day of VMT Release after the therapy
1	64	F	phakic	no		372	20/160	20/80	yes	14
2	69	F	phakic	no	682		20/63	20/50	yes	28
3	68	F	phakic	no	408		20/40	20/25	yes	7
4	67	F	phakic	yes	337		20/20	20/20	yes	28

*Bas* baseline

*Dia* diameter

*VMA* vitreomacular adhesion

*BCVA* best corrected visual acuity

*VMT* vitreomacular traction

*MH* macular hole

*F* female

*μm* micrometer

**Fig. 1:**
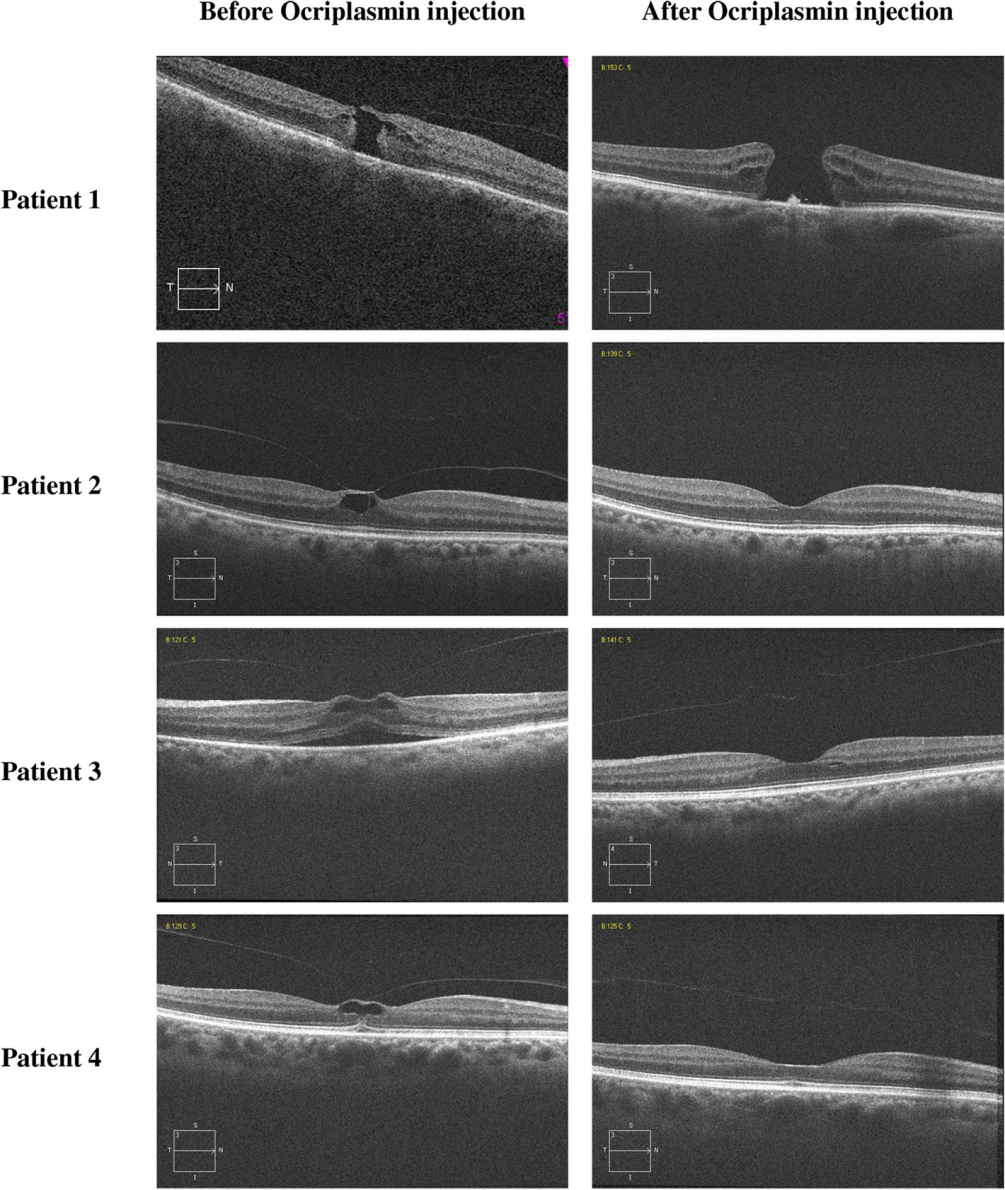
Baseline and post-operative OCT scans (after six months) in all treated subjects. The selected OCT scans show the release of VMT post-treatment in all patients. In patient 1, we observed an enlargement of the full-thickness macular hole

We observed a VMT non-surgical resolution in all patients as shown by Fig. 1, which reports the OCT scans before and after VMT resolution at six months post-treatment. VMT release occurred within 1 week in patient 3, within 2 weeks in patient 1, and within 4 weeks in patient 2 and patient 4. Moreover, we observed a significant decreasing trend (*p* < 0.001) in the foveal thickness, assessed on foveal OCT scans, at an estimated linear rate of −44.3 μm per year. However, the Ocriplasmin injection did not achieve the closure of the MH in patient 1, which worsened from stage 2 to stage 4. Except patient 1, we did not observe disruption of the photoreceptor inner segment-outer segment ellipsoid in any of the OCT scans after the injection of Ocriplasmin.

**Fig. 2:**
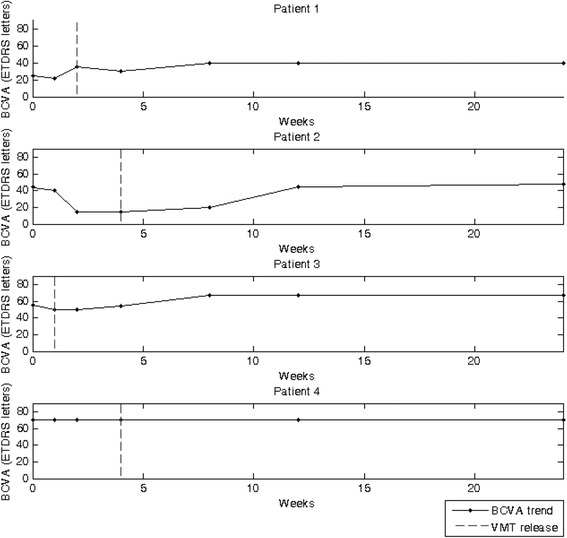
Time courses of BCVA over the 6-month follow-up. In the first three patients BCVA, following an initial decline, improved after the release of VMT; in the last patient the BCVA remained stable (20/20) over all the follow-up

In the treated eyes, the average BCVA increased from 48 ± 19 ETDRS letters (equivalent to 20/63) at baseline to 56 ± 14 ETDRS letters (equivalent to 20/40) at the six month post-treatment time-point, whereas BCVA remained stable in the untreated eyes (average at baseline and the last time-point: 36 ± 23 ETDRS letters, equivalent to 20/100). In particular, in the first three patients, we observed an improvement of BCVA in the treated eyes of at least one ETDRS line (six months), while in the fourth patient BCVA was of 10/10 at the baseline and remained stable over the follow-up and metamorphopsia disappeared. The longitudinal statistical analysis showed a significant improvement of BCVA in the treated eyes of about 0.975 letters/week (*p* = 0.033), while BCVA remained stable in the untreated eyes (baseline: 36 ± 22 ETDRS letters; last time-point: 35 ± 23 ETDRS letters; *p* = 0.25). In particular, we observed that BCVA declined after injection (average: 4 ± 1 ETDRS letters) and started to improve after the resolution of VMT (Fig. 2). We observed the smallest BCVA improvement (4 ETDRS letters) in patient 2, who required the longest time for VMT release (1 month).

Macular sensitivity, assessed by MP1, increased only in the treated eyes of two patients, 2 and 4 (Table 2), while fixation stability improved in three patients. The longitudinal analysis showed no significant difference in macular sensitivity in both treated (baseline: 12.7 ± 6.9 dB; last time-point: 11.3 ± 8.3 dB; *p* = 0.16) and untreated eyes (baseline: 10.3 ± 5.0 dB; last time-point: 11.4 ± 3.1 dB; *p* = 0.81). As regards fixation stability, a significant improvement of the percentage of fixation points within the 4° circle was assessed in the treated eyes (baseline: 57.8 ± 45.3 %; last time-point: 81.8 ± 20.8 %; β = 0.39; *p* = 0.029), even if the trend of increased percentage of fixation points within the 2° circle was not significant (baseline: 38.7 ± 41.7 %; last time-point: 57.7 ± 34.1 %; β = 1.03; *p* = 0.061).

**Table 2:** Comparison of micriperimetric features between baseline and the last post-treatment time-points

Id	Treated eyes								Untreated eyes							
	Baseline				Last time-point				Baseline				Last time-point			
	MS dB	FS2 %	FS4 %	Fixation stability	MS dB	FS2 %	FS4 %	Fixation stability	MS dB	FS2 %	FS4 %	Fixation stability	MS dB	FS2 %	FS4 %	Fixation stability
1	19.4	14	44	Instable	15.3	47	83	Relatively stable	8	69	96	Relatively stable	8	23	63	Unstable
2	6.4	94	100	Stable	10.2	92	97	Stable	15.9	96	99	Stable	15	93	97	Stable
3	7.3	0	0	Instable	0.4	77	95	Stable	4.6	83	94	Stable	9.8	66	94	Relatively stable
4	18	47	87	Relatively stable	19.6	15	52	Instable	12.6	43	88	Relatively stable	12.5	33	77	Relatively stable

*MS* macular sensitivity

*FS2* percentage of fixation points within the 2° circle

*FS4* percentage of fixation points within the 4° circle

*dB* decibel

**Fig. 3:**
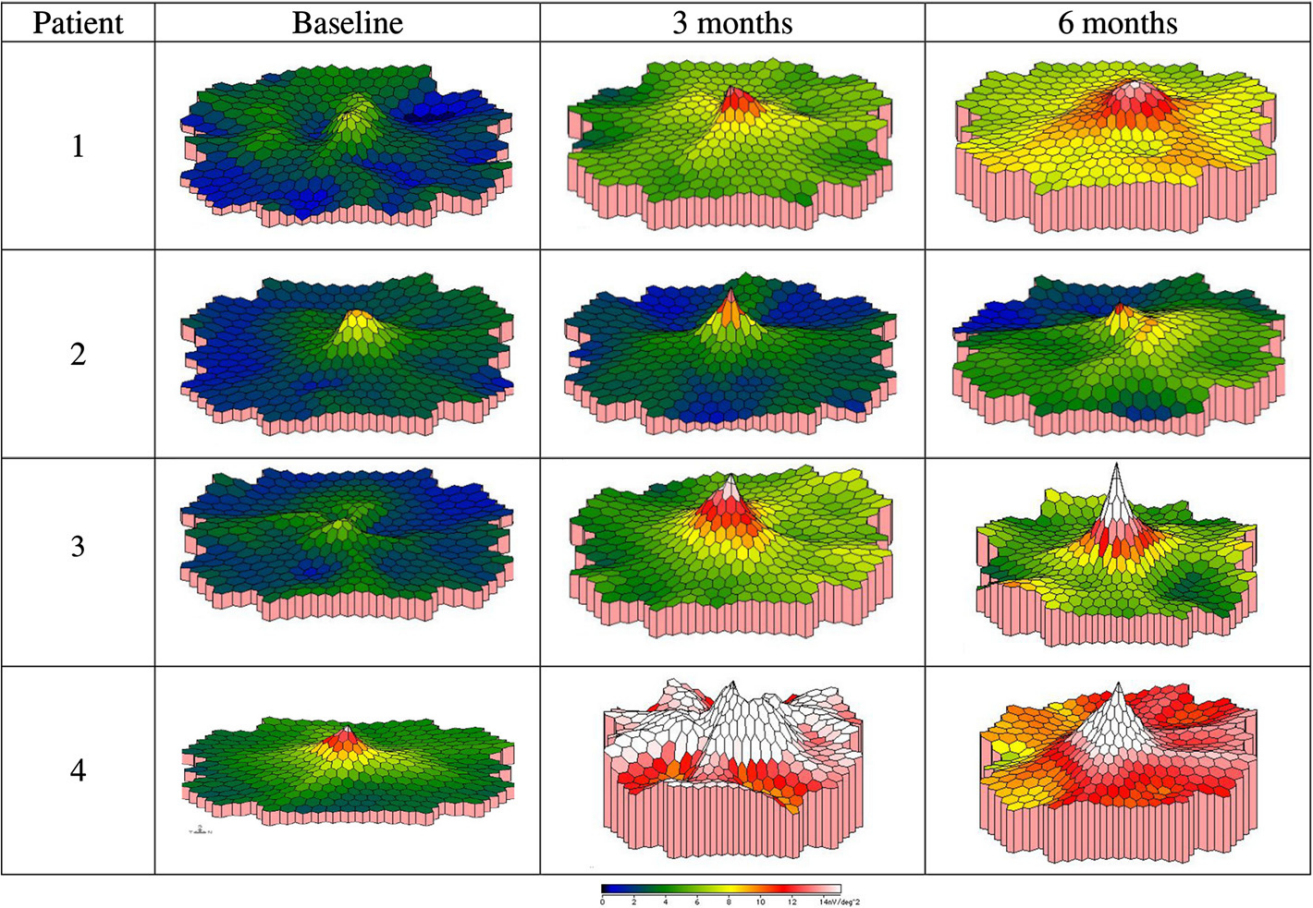
Baseline and postoperative mfERG examinations in all treated subjects. The first order response density of mfERG examinations at baseline and at successive time-points revealed a progressive increased foveal peak response in the treated eyes over the follow-up

mfERG revealed a progressive increased foveal peak response in the treated eyes over the follow-up, as shown in the first order response density at baseline and at successive time-points, reported in Fig. 3. Additional file 1 reports the data of mfERG responses at the different time-points. The longitudinal analysis, reported in Table 3, showed a significant decrease of N1 implicit time in all the rings but the foveal one, of N1 amplitude in the first two rings, of P1 implicit time in the first three rings, and a significant increase of P1 amplitude in the first two rings. Finally, we observed a significant relationship between BCVA and mfERG responses, in particular: a positive correlation with amplitude of P1 peak (β = 1.406; *p* < 0.001) and amplitude of N1 peak (β = 1.302; *p* < 0.001); and a negative correlation with the P1 implicit time (β = −0.088; *p* = 0.004).

**Table 3:** Longitudinal regression analysis of mfERG responses

Ring	N1 implicit time			N1 amplitude			P1 implicit time			P1 amplitude		
	β	Std. Err.	*p*-value	β	Std. Err.	*p*-value	β	Std. Err.	*p*-value	β	Std. Err.	*p*-value
1	.074	.0467	.111	**−1.344**	**.2415**	**<.001**	**-.238**	**.0548**	**<.001**	**2.558**	**.5020**	**<.001**
2	**-.227**	**.0541**	**<.001**	**-.576**	**.2467**	**.019**	**-.110**	**.0519**	**.034**	**1.093**	**.5034**	**.030**
3	**-.267**	**.0521**	**<.001**	-.217	.2523	.389	**-.225**	**.0565**	**<.001**	.458	.5113	.371
4	**-.178**	**.0534**	**.001**	.016	.2534	.948	-.050	.0572	.380	.043	.5102	.933
5	**-.264**	**.0500**	**<.001**	-.049	.2546	.848	**-.234**	**.0537**	**<.001**	-.140	.5064	.782
6	**-.191**	**.0465**	**<.001**	.053	.2546	.835	-.106	.0577	.067	-.053	.5115	.918

*Std. Err* Standard Error

Significant p-value and related data (i.e, coefficient and standard error) are in bold

## Discussion

Intravitreal injection of Ocriplasmin represents a novel treatment option supplementing observation and vitrectomy in the management of patients with symptomatic vitreomacular interface disorders. In our study, we report a case series of four patients treated with injection of Ocriplasmin and we evaluate the release of VMT not only from the morphological but also functional standpoint. We observed an overall incidence of successful VMT release (100 %), that was much higher compared to the results of the MIVI-TRUST (MIVI-006, MIVI-007) clinical trials (26.5 %) [1]. This could be explained by the accurate selection of the patients with three independent characteristics, which were shown to be associated with a more favourable outcome, i.e., absence of epiretinal membrane, adhesion diameter less than 1,500 μm, presence of natural lens in the treated eye. In addition, we show the clinical results of a patient who presented BCVA higher than 20/25 before injection.

Although this was a small series with a limited follow-up interval, no major adverse effects, except the worsening of the macular hole and the associated disruption of photoreceptor inner segment–outer segment in the patient 1, were encountered following intravitreal injection of Ocriplasmin, specifically, no retinal tears, retinal detachment, post-injection inflammation, acute severe panretinal dysfunction. The development or worsening of macular hole was the most frequent eye serious adverse event reported in Ocriplasmin trials [1], with no significant difference between treatment and control group. Unfortunately, in our treated patient with MH, in spite of VMT release, MH enlarged and changed from stage 2 to stage 4. However, an improvement in visual function and mfERG response was observed and may be due to the increased activity of photoreceptors in the perilesional area (corresponding to ring 1 and 2) after the release of VMT.

Similarly to the findings on the patients receiving Ocriplasmin injection in clinical trials and recent reports of treated cases [3, 4, 14], we observed a decline in BCVA after Ocriplasmin injection, followed by an improvement of at least one ETDRS line, which started after VMT resolution, even if associated with vitreous floaters and photopsia. No other symptoms, e.g. the discromatopsia, which was reported in some cases by other authors [3, 4, 14], were reported by our patients. Furthermore, in our study, for the first time in the literature, we evaluated the macular functionality through the study of tests such as MP1 and mfERG for a six month follow-up.

MP1 enabled to observe an improvement in fixation stability, even if with stable macular sensitivity. The analysis of mfERG responses showed a significant increase of N1 and P1 amplitudes after Ocriplasmin injection (six-month follow-up). The mfERG waveforms originate from cone photoreceptors and bipolar and Muller cells respectively [15]. Hence, our findings, through the use of mfERG, allow to highlight the functional improvement of retinal layers, induced by the injection of Ocriplasmin. Moreover, we compared the time course of visual acuity and the responses of mfERG, also in relationship with the time of the VMT release. This analysis showed that there is a statistically significant correlation between the resolution of the VMT, the onset of BCVA increase and of improved mfERG responses. Therefore, the longitudinal analysis shows that visual acuity changes are correlated with the change in mfERG responses. Moreover, the results of our study demonstrate that the morphological resolution with the release of VMT does not coincide with an immediate functional recovery. In fact, functional tests, as mfERG and visual acuity measurement, also after VMT release, improved progressively throughout the course of follow-up. Probably, it takes longer to restore the architecture of retinal layers and their functionality with respect to VMT release. Limited evidence has been reported in literature about the restoration time also after vitrectomy for VMT release, however a recent meta-analysis demonstrated a mean improvement of 0.28 LogMAR in BCVA after vitrectomy [16], which is comparable with the mean improvement in BCVA that we observed in our patients with BCVA reduction (0.21 LogMAR). Moreover, the follow-up time is usually longer than six months in the studies investigating vitrectomy, suggesting that the surgical procedure requires a longer time for restoration of visual functionality.

## Conclusions

Our study shows that Ocriplasmin was safe and effective in the treatment of VMT not associated with MH, since it leaded not only to a morphological recovery but mostly to an improvement of macular functionality within a six-month follow-up. However, the patients should be selected carefully for Ocriplasmin treatment (e.g., absence of epiretinal membrane, adhesion diameter less than 1,500 μm, presence of natural lens in the treated eye) and monitored cautiously using SD-OCT and other functional techniques (i.e., mfERG and MP1). However, the treatment of MH is still challenging because of the risk of its enlargement even in case of VMT release (primary endpoint) not associated to hole closure (secondary endpoint). Further studies could assess the improvement of visual function in the treatment of VMT associated with MH by Ocriplasmin, also in the case of non-closure of the macular lesion. Finally, follow-up visits should exceed 12 weeks after injection, since long-term effects need to be investigated in order to evaluate the safety and effectiveness of Ocriplasmin.

## Additional file

Additional file 1: Multifocal electroretinogram data. (XLSX 14 kb)
